# GLTSCR2 promotes the nucleoplasmic translocation and subsequent degradation of nucleolar ARF

**DOI:** 10.18632/oncotarget.9957

**Published:** 2016-04-28

**Authors:** Sun Lee, Young-Eun Cho, Sang-Hoon Kim, Yong-Jun Kim, Jae-Hoon Park

**Affiliations:** ^1^ Department of Pathology, College of Medicine, Kyung Hee University, Seoul 130-701, Korea

**Keywords:** glioma tumor suppressor candidate region gene 2 protein, alternative reading frame, subcellular localization, degradation, translocation

## Abstract

The alternative reading frame protein (p14ARF/ARF) is a key determinant of cell fate, acting as a potent tumor suppressor through a p53/MDM2-dependent pathway or promoting apoptosis in a p53-independent manner. The ARF protein is mainly expressed in the nucleolus and sequestered by nucleophosmin (NPM), whereas ARF-binding proteins, including p53 and MDM2, predominantly reside in the nucleoplasm. This raises the question of how nucleolar ARF binds nucleoplasmic signaling proteins to suppress tumor growth or inhibit cell cycle progression. GLTSCR2 (also known as PICT-1) is a nucleolar protein involved in both tumor suppression and oncogenesis in concert with p53, NPM, and/or MYC. Here, we show that GLTSCR2 increases nucleoplasmic ARF translocation and its degradation. Specifically, GLTSCR2 bound to ARF, and GLTSCR2-ARF complexes were released to the nucleoplasm, where GLTSCR2 increased the binding affinity of ARF for ULF/TRIP12 (a nucleoplasmic E3-ubiquitin ligase of ARF) and enhanced ARF degradation through the polyubiquitination pathway. Our results demonstrate that nucleolar/nucleoplasmic GLTSCR2 is a strong candidate for promoting the subcellular localization and protein stability of ARF.

## INTRODUCTION

Glioma tumor suppressor candidate region gene 2 protein (GLTSCR2) is a nucleolar protein that translocates between the nucleolus and nucleoplasm and is involved in positively and negatively regulating the stability of p53 and nucleophosmin (NPM), respectively [1, 2].

The alternative reading frame (p14ARF/ARF) protein is a key regulator of cell proliferation and death [3]. In response to oncogenic activation, ARF is upregulated and can induce cell cycle arrest in p53-dependent and -independent manners [4]. Specifically, ARF binds with and inactivates the E3 ubiquitin ligase HDM2, a primary negative regulator of p53, to inhibit p53 degradation through the ubiquitination-proteasome pathway [5, 6]. However, most ARF is found within the nucleolus, whereas the ARF-binding proteins p53 and HDM2 predominantly localize to the nucleoplasm. Therefore, because of this discrepancy in the subcellular localization of ARF, p53, and HDM2, it is unclear how nucleolar ARF participates in p53 signaling. One hypothesis is that HDM2 sequestration by ARF in the nucleolus blocks the binding between HDM2 and p53 [7, 8]. Alternatively, trafficking of ARF between the nucleolus and nucleoplasm may occur; indeed, a complex composed of ARF, HDM2, and p53 has been detected in the nucleoplasm [9]. Moreover, disruption of binding between ARF and NPM, a nucleolar binding partner of ARF, triggered the nucleoplasmic redistribution of nucleolar ARF, allowing ARF to bind with p53 [10]. In contrast to nucleoplasmic ARF, nucleolar ARF is involved in ribosomal biogenesis [11]. Overexpression of ARF interferes with ribosomal RNA processing in a p53-independent manner, possibly by binding to and inhibiting nucleolar ribosomal RNA biogenesis factors, including NPM [12, 13]. These findings indicate that the subnuclear compartmentalization of ARF and its expression level are crucial for defining the cellular activity of ARF. However, the molecular mechanisms regulating the expression of nucleolar ARF and nucleolar-nucleoplasmic trafficking mechanisms are not yet clear.

Here, we report the binding between GLTSCR2 and ARF and the effects of this binding on the intranuclear localization and stability of ARF. Our findings demonstrated that GLTSCR2 was a key molecule involved in the nucleolus-nucleoplasmic protein axis, which is involved in cancer development and/or progression.

## RESULTS

### GLTSCR2 binds to ARF

**Figure 1:**
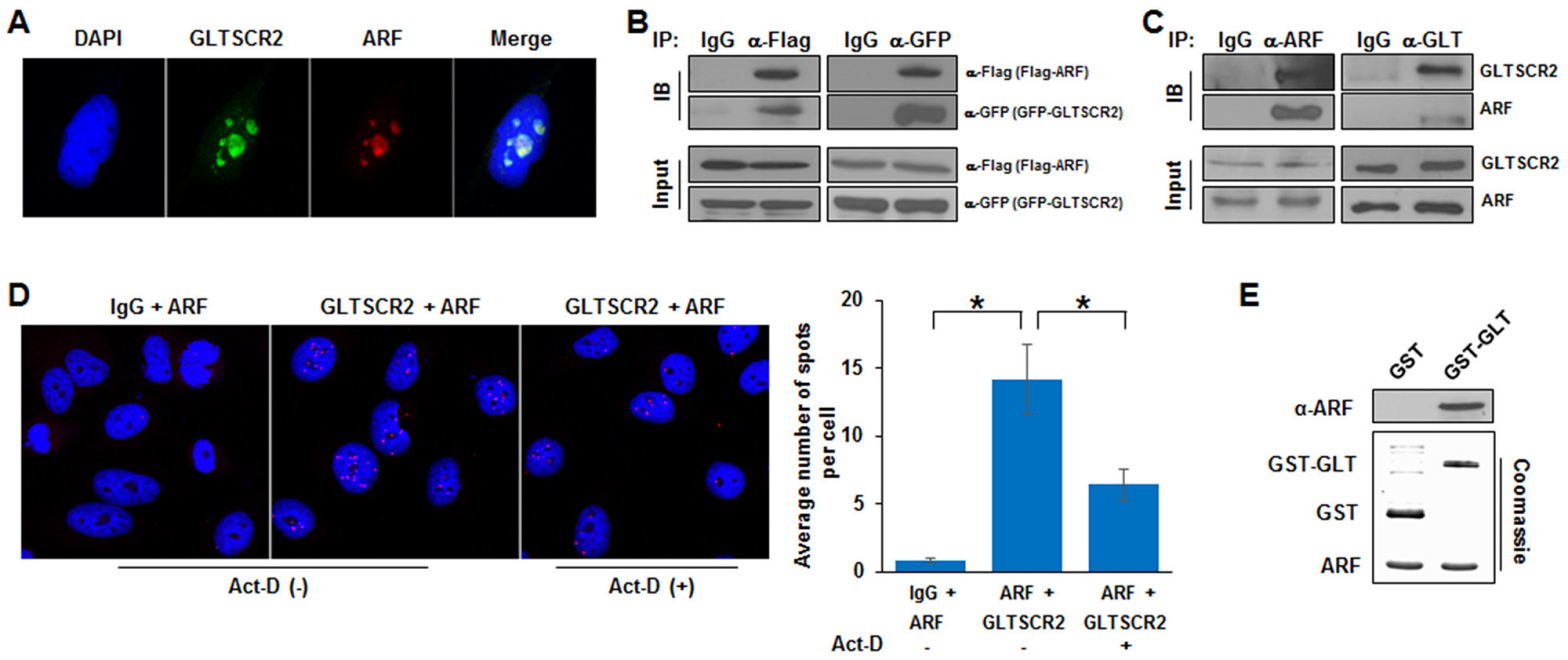
GLTSCR2 binding to ARF **A**. HeLa cells were co-immunostained with anti-GLTSCR2 and anti-ARF antibodies. Cells were viewed under a fluorescence microscope after DAPI staining (original magnification, 600×). **B**. HEK-293T cells were transfected with plasmids expressing Flag-ARF and GFP-GLTSCR2 for 24 h. Cell lysates were immunoprecipitated with anti-Flag (left panel) or anti-GFP (right panel) antibodies, and precipitates were subjected to immunoblotting using the indicated antibodies. An isotype-matched control IgG was also used for immunoprecipitation. A 10% loading control is shown in the lower panel. **C**. Cell lysates were immunoprecipitated with anti-ARF (left panel) or anti-GLTSCR2 (right panel) antibodies, and precipitates were subjected to immunoblotting using the indicated antibodies. **D**. HeLa cells were untreated or treated with 100 μM actinomycin D (Act-D) for 5 h, and then a PLA was performed using rabbit anti-GLTSCR2 and mouse anti-ARF antibodies. Representative PLA images are shown in the left panel. IgG was used as a control antibody. The numbers of spots in at least 100 cells were counted; the average numbers of spots per cell is shown in the right panel. *p < 0.01. **E**. A total of 200 ng of purified GST alone or GST-tagged GLTSCR2 immobilized on glutathione beads was incubated with 200 ng of recombinant ARF protein, which was purified from bacteria and cleaved with thrombin. Bound ARF was detected with an anti-ARF antibody (upper panel). Recombinant GST, GST-GLTSCR2, and ARF proteins were visualized by Coomassie blue staining (lower panel).

Physical interactions and nucleolus-nucleoplasmic trafficking of nucleolar proteins are critical for establishing the nucleolar-nucleoplasmic axis, which has been shown to regulate oncogenic responses [14]. Initially, to compare the subcellular localization of GLTSCR2 and ARF, we performed co-immunocytochemical staining of HeLa cells using anti-GLTSCR2 and anti-ARF antibodies. As shown in Figure 1A, the expression of both endogenous GLTSCR2 and ARF were predominantly nucleolar and showed co-localization, suggesting that these proteins may bind each other. To investigate whether GLTSCR2 and ARF bind to each other, HEK-293T cells were co-transfected with plasmids expressing GFP-tagged GLTSCR2 (GFP-GLT) and Flag-tagged ARF (Flag-ARF), and immunoprecipitation and immunoblotting assays were performed. Using anti-Flag antibodies, Flag-ARF was found to associate with GFP-GLT (Figure 1B, left panel). Conversely, GFP-GLT specifically immunoprecipitated with Flag-ARF using an anti-GFP antibody (Figure 1B, right panel), indicating that GLTSCR2 and ARF bound each other. Binding specificity between GFP-GLTR2 and Flag-ARF was confirmed by immunoprecipitation using GFP-human growth and transformation-dependent protein (HGTDP) and Flag-ARF (Supplementary Figure S1). To exclude the possibility of promiscuous binding due to protein overexpression, we performed immunoprecipitation without ectopic expression of ARF or GLTSR2, using specific antibodies. As shown in Figure 1C, endogenous GLTSCR2 and ARF bound to each other. Protein binding between GLTSCR2 and ARF was further confirmed by performing proximity ligation assays (PLAs) (Figure 1D). Next, we performed *in vitro* pull-down assay to determine whether protein binding between GLTSCR2 and ARF was direct or involved an additional cellular partner. As shown in Figure 1E, recombinant GLTSCR2 protein was pulled down directly with ARF *in vitro*.

It has been reported that nucleolar stress induces nucleoplasmic translocation of both GLTSCR2 and ARF [15, 16]. Thus, to characterize the protein binding between GLTSCR2 and ARF under nucleolar stress, we performed PLAs in HeLa cells treated with actinomycin D (Act-D). Interestingly, nucleolar stress induced by Act-D decreased GLTSCR2 binding to ARF (Figure 1D). Together, our results showed that GLTSCR2 and ARF directly bound to each other and that their binding affinities decreased during nucleolar stress.

### C-terminal region of ARF bound to the central region of GLTSCR2

**Figure 2:**
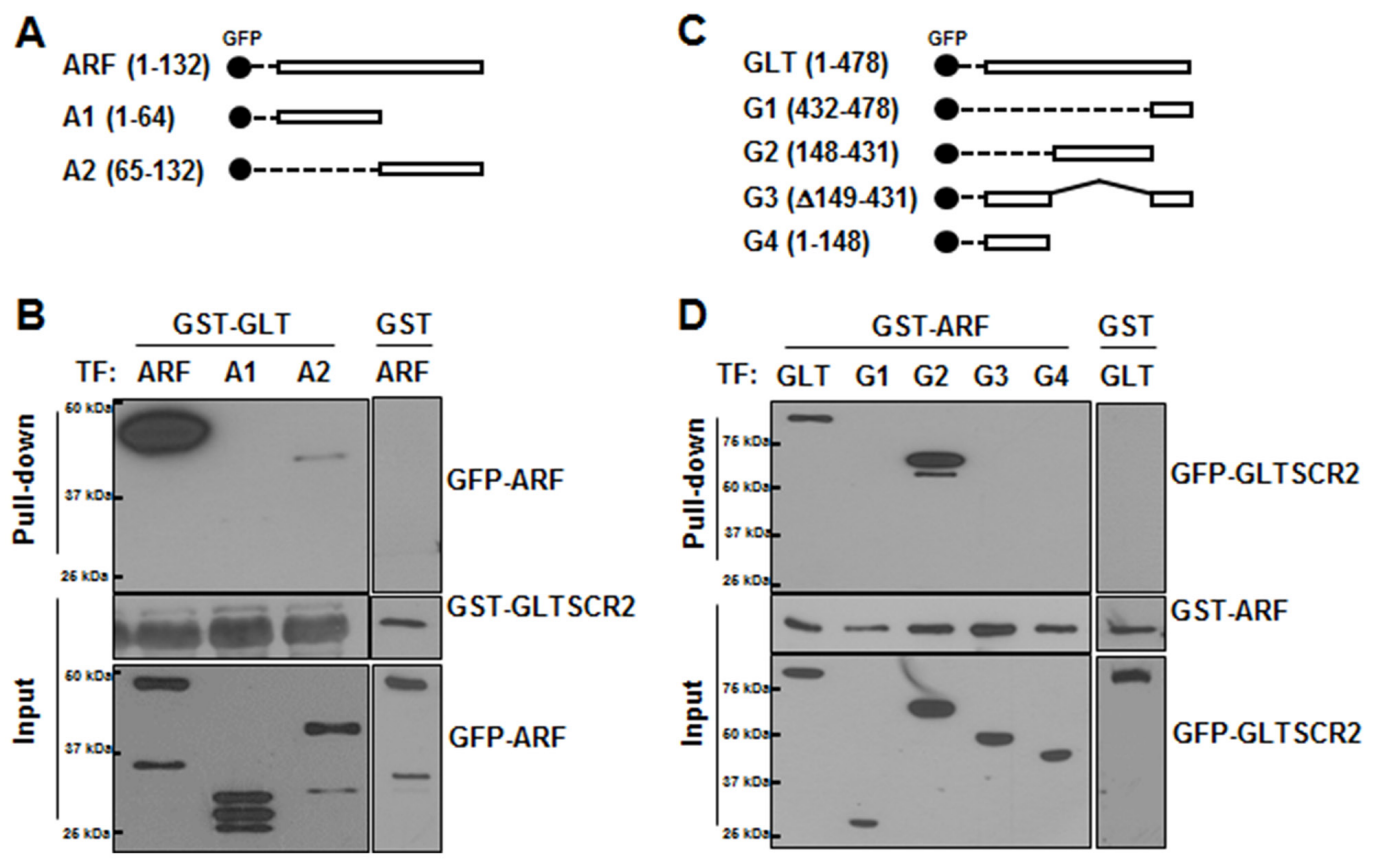
C-terminal region of ARF bound to the central region of GLTSCR2 **A**. Schematic diagram of GFP-tagged wild-type or splicing-mutant plasmids of ARF. **B**. HEK-293T cells were transfected with GFP-tagged ARF-, A1-, or A2-expressing plasmids for 24 h. Then, 1 μg of recombinant GLTSCR2-GST fusion protein (left panel) or GST protein (right panel) was added to the cell lysates, and pull-down assays were performed. Precipitates from the pull-down assays were subjected to immunoblotting using anti-GFP antibodies (upper panel). Loading controls are shown in the middle and lower panels. **C**. Schematic diagram of GFP-tagged wild-type or splicing-mutant plasmids of GLTSCR2. **D**. HEK-293T cells were transfected with plasmids expressing GFP-tagged wild-type GLTSCR2 or the indicated GLTSCR2 mutants for 24 h, and pull-down assays after adding ARF-GST (left panel) or GST (right panel) were then performed, as described for (B). Precipitates from the pull-down assays were subjected to immunoblotting using anti-GFP antibodies (upper panel). Loading controls are shown in the middle and lower panels.

To map the binding domains between GLTSCR2 and ARF, HEK-293T cells were transfected with plasmids encoding GFP-tagged wild-type ARF or mutant ARFs (Figure 2A), and the cell lysates were subjected to GST pull-down assays using a GST-GLTSCR2 fusion protein. GLTSCR2-ARF complexes were then analyzed by western blotting. GLTSCR2 was co-precipitated with wild-type ARF or the C-terminal region (amino acids 65–132) of ARF, but not with the N-terminus (amino acids 1–64) of ARF (Figure 2B). Similarly, cells were transfected with a series of truncation mutants of GFP-tagged GLTSCR2 (Figure 2C) and then subjected to GST pull-down assays using the GST-ARF fusion protein. As shown in Figure 2D, the central portion of GLTSCR2 (amino acids 148–431) was required for ARF binding. Nonspecific binding between GST and ARF or GLTSCR2 was not detected (Figure 2B and Figure 2D, right panel).

### GLTSCR2 induced nucleoplasmic translocation of nucleolar ARF

**Figure 3:**
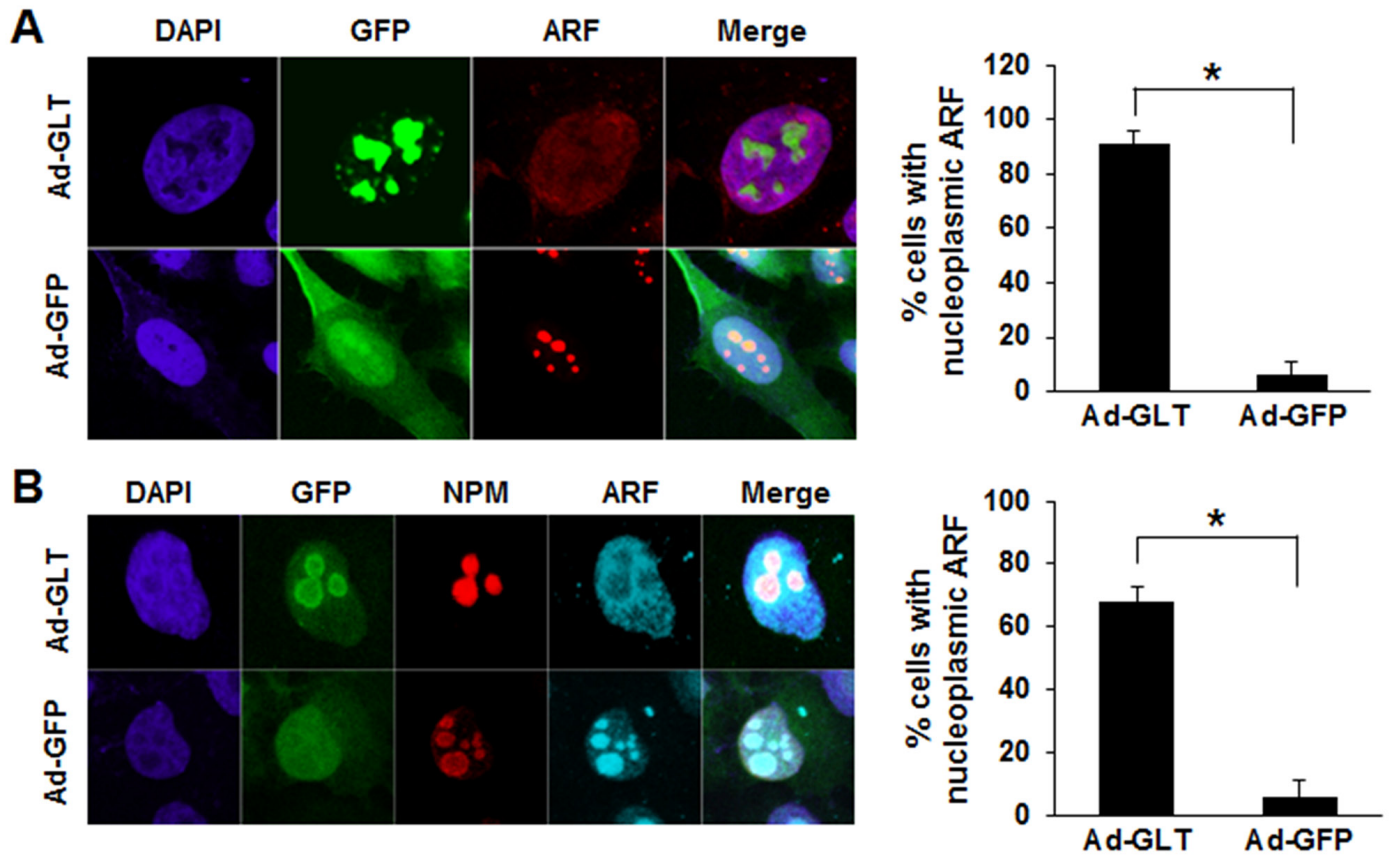
GLTSCR2-induced nucleoplasmic redistribution of ARF **A**. HeLa cells were transduced with Ad-GLT or Ad-GFP adenovirus constructs for 24 h and then immunostained with anti-ARF antibodies. Representative images are shown in the left panel (original magnification, 600×). Cells with nucleoplasmic ARF were counted among at least 200 cells following Ad-GLT or Ad-GFP transduction under a fluorescence microscope (right panel). Data from 3 independent experiments are shown as the means ± SDs; *p < 0.01. **B**. HeLa cells stably transfected with V5-tagged nucleophosmin (NPM)-expressing plasmids were transduced with Ad-GLT or Ad-GFP for 24 h. Cells were then co-immunostained using Texas-Red conjugated anti-V5 or Cy5-conjugated anti-ARF antibodies. Representative images are shown in the left panel (original magnification, 600×). Cells with nucleoplasmic ARF were counted among at least 200 cells following Ad-GLT or Ad-GFP transduction under a fluorescence microscope (right panel). Data from 3 independent experiments are shown as the means ± SDs; *p < 0.01.

ARF is predominantly localized in the nucleolus in high-molecular-weight complexes with NPM, and formation of the ARF-NPM complex in the nucleolus enhances the stability of ARF [17]. Previously, we reported that GLTSCR2 binds to NPM to facilitate the nucleoplasmic redistribution of NPM [2]. Because ARF-NPM binding is required for the nucleolar localization of ARF, we hypothesized that GLTSCR2 may inhibit the nucleolar localization of ARF. Thus, we transduced HeLa cells with a GFP-tagged GLTSCR2-expressing (Ad-GLT) or empty (Ad-GFP) adenovirus and then determined the localization of ARF using immunocytochemistry. As shown in Figure 3A, ectopic expression of GLTSCR2 facilitated the nucleoplasmic redistribution of ARF, which was not observed in control cells transduced with Ad-GFP. However, it is unclear whether the increased nucleoplasmic localization of ARF resulted from release from the nucleolus or a decreased import of newly synthesized ARF to the nucleolus. Therefore, to elucidate the mechanism underlying GLTSCR2-dependent nucleoplasmic ARF localization, we transduced HeLa cells with Ad-GFP or Ad-GLT, treated the cells with cycloheximide, and then determined the proportion of cells with nucleoplasmic ARF. As shown in Supplementary Figure 2, cycloheximide did not affect the nucleoplasmic redistribution of ARF in GLTSCR2-overexpressing cells, indicating that nucleoplasmic ARF accumulation resulted from increased release from the nucleolus and not from a transport failure of newly synthesized ARF to the nucleolus. Next, to investigate whether GLTSCR2-induced translocation of ARF was increased by NPM degradation [2], GLTSCR2 was transiently overexpressed in HeLa cells stably overexpressing NPM, and the localization of ARF was determined using immunocytochemistry. As shown in Figure 3B, inhibition of GLTSCR2-mediated NPM degradation did not impair ARF release from the nucleolus, indicating that the nucleoplasmic translocation of ARF by GLTSCR2 did not result from GLTSCR2-mediated NPM degradation.

### The binding between GLTSCR2 and ARF was crucial for the nucleoplasmic translocation of ARF

**Figure 4:**
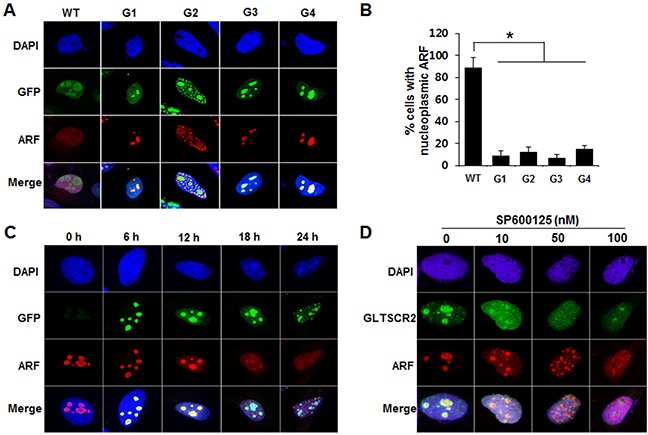
GLTSCR2-ARF binding was crucial for the nucleoplasmic translocation of ARF **A**. HeLa cells were transfected with plasmids expressing GFP-tagged wild-type or one of several mutant GLTSCR2 plasmids for 24 h, followed by immunostaining with anti-ARF antibodies. Original magnification, 600×. **B**. Cells were transfected as described in (A), and transfected cells with nucleoplasmic ARF were counted under a fluorescence microscope. Data from 3 independent experiments are shown as means ± SDs; *p < 0.01. **C**. Cells were transduced with an Ad-GLT viral vector in the presence of 1 ng doxycycline for 24 h. Subsequently, doxycycline was removed, and immunocytochemical staining was performed using an anti-ARF antibody at the indicated time points. **D**. Cells were treated with the indicated concentrations of SP600125 for 6 h, followed by co-immunostaining with anti-GLTSCR2 and anti-ARF antibodies.

To investigate the role of GLTSCR2-ARF binding in GLTSCR2-mediated ARF translocation, we transfected cells with a plasmid expressing wild-type GLTSCR2 or one of several GLTSCR2 mutants and then analyzed ARF localization. As shown in Figure 4A and 4B, wild type GLTSCR2 and the G2 mutant induced nucleoplasmic ARF translocation, while the G1, G3, and G4 mutants did not. Interestingly, the G2 mutant, which has an ARF-binding domain, did not induce diffuse nucleoplasmic ARF redistribution. Instead, ARF showed a granular nucleoplasmic pattern with complete colocalization with the G2 mutant in the nucleoplasm (Figure 4A and Supplementary Figure S3). Although these findings indicate that GLTSCR2-ARF binding was crucial for nucleoplasmic ARF translocation, it was unclear how GLTSCR2-ARF binding in the nucleolus induced the nucleoplasmic translocation of ARF, rather than promoting its sequestration in the nucleolus. Thus, we hypothesized that ARF translocates in complex with GLTSCR2 when GLTSCR2 is released to the nucleoplasm, considering that GLTSCR2 shifts between the nucleolus and nucleoplasm in response to various cellular stresses [15]. To test this hypothesis, we examined whether ARF release from the nucleolus is induced when nucleoplasmic GLTSCR2 is increased by ectopic GLTSCR2 overexpression. Ectopic GLTSCR2 expression was induced in HeLa cells by transduction with a doxycycline-inducible Ad-GLT (using the Tet/OFF system), and its localization was subsequently determined after changing the medium in separate cultures every 6 h to remove doxycycline at progressively later time points. As shown in Figure 4C and Supplementary Figure 4, we found that increased nucleoplasmic GLTSCR2 expression paralleled concomitant ARF release. Next, we investigated whether endogenous ARF could be translocated when GLTSCR2 shifted to the nucleoplasm, without overexpression. Cells were treated with SP600125 because c-Jun N-terminal kinase (JNK) activity is required for nucleolar GLTSCR2 localization [18], and endogenous ARF localization was determined. As shown in Figure 4D, GLTSCR2 shifting to the nucleoplasm following JNK inhibition was concomitant with nucleoplasmic ARF redistribution. Taken together, our findings demonstrated that nucleolar ARF translocates to the nucleoplasm by GLTSCR2 in a GLTSCR2-ARF complex.

### GLTSCR2 enhanced the degradation of ARF

**Figure 5:**
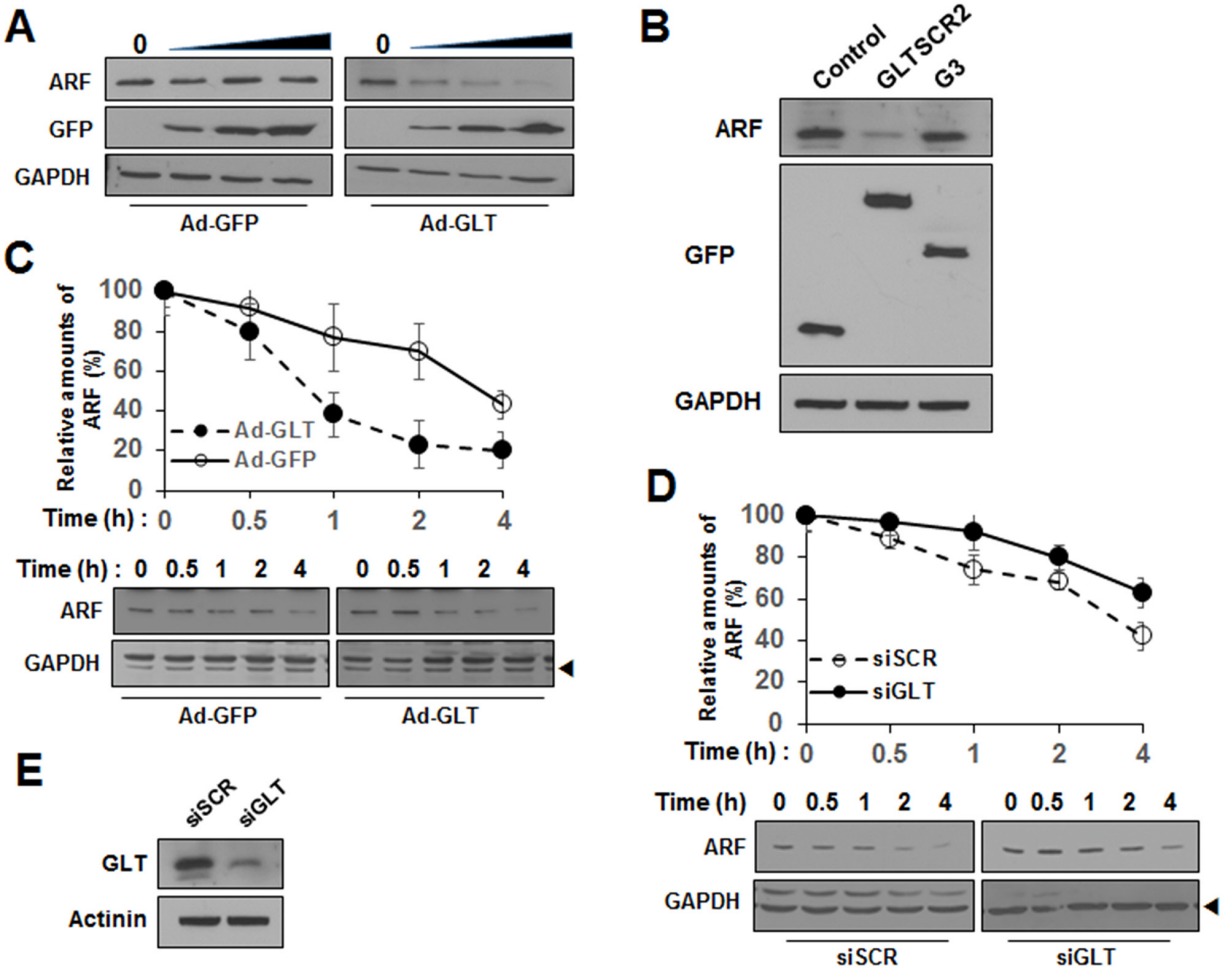
GLTSCR2 enhanced the degradation of ARF **A**. HeLa cells were transduced with increasing MOIs of Ad-GFP or Ad-GLT for 24 h, and lysates were subjected to immunoblotting using the indicated antibodies. **B**. Cells were transfected with the indicated plasmids for 24 h, and lysates were analyzed by immunoblotting using the indicated antibodies. **C**. HeLa cells were transduced with Ad-GLT or Ad-GFP for 12 h. After treatment with 100 μg/mL cycloheximide, cell lysates were prepared at the indicated time points (0–4 h). ARF expression was studied by immunoblotting after normalization to GAPDH expression. The plot shows densitometric quantification of cycloheximide chase assays (upper panel). The data shown represent the percentages of ARF intensity from 3 independent experiments compared with that at the 0-h time point. Representative immunoblot images of cycloheximide chase assays are shown in the lower panel. **D**. Cells were transfected with siRNA targeting GLTSCR2 (siGLT) or scrambled siRNA (siSCR) for 2 days, followed by treatment with 100 μg/mL cycloheximide. Cells were harvested at the indicated time points, and cycloheximide chase assays were performed. **E**. Western blot images showing GLTSCR2 expression in the cells used in (D).

Previous data have shown that the non-nucleolar form of ARF is susceptible to proteasomal degradation [17]. Thus, our results showing that GLTSCR2 facilitated the nucleoplasmic translocation of ARF led us to speculate that GLTSCR2 may participate in ARF degradation. To test this, HeLa cells were transduced with increasing multiplicities of infection (MOIs) of either Ad-GFP or Ad-GLT, and the expression levels of ARF were determined at 24 h after viral transduction. As shown in Figure 5A, ectopic expression of GLTSCR2 downregulated the expression of ARF in a GLTSCR2-dependent manner, whereas transduction with Ad-GFP did not alter ARF expression. In addition, GLTSCR2 induced ARF degradation through the proteasomal pathway (Supplementary Figure S5). Interestingly, however, the GLTSCR2-nonbinding G3 mutant did not alter the ARF expression level (Figure 5B). To elucidate the mechanisms through which GLTSCR2 downregulated ARF, we examined the protein stability of ARF using cycloheximide chase assays. HeLa cells were treated with cycloheximide, and the half-life of ARF was determined by densitometric analysis. The half-life was approximately 4 h in GFP-expressing control cells, but reduced to less than 1 h in GLTSCR2-expressing cells (Figure 5C). However, *ARF* mRNA levels remained constant after GLTSCR2 overexpression (data not shown). Interestingly, half-life of ARF was not reduced by GLTSCR2-nonbinding G3 overexpression (Supplementary Figure S6). Next, we performed chase assays in GLTSCR2-knockdown cells. GLTSCR2 expression was downregulated by transfecting HeLa cells with small-interfering RNA targeting GLTSCR2 (siGLT). Cycloheximide chase assays revealed the reduced degradation of ARF in GLTSCR2-knockdown cells as compared with that in control cells transfected with scrambled siRNA (siSCR; Figure 5D and 5E). Taken together, our results showed that GLTSCR2 enhanced the degradation of ARF.

### GLTSCR2 promoted the polyubiquitination of ARF

**Figure 6:**
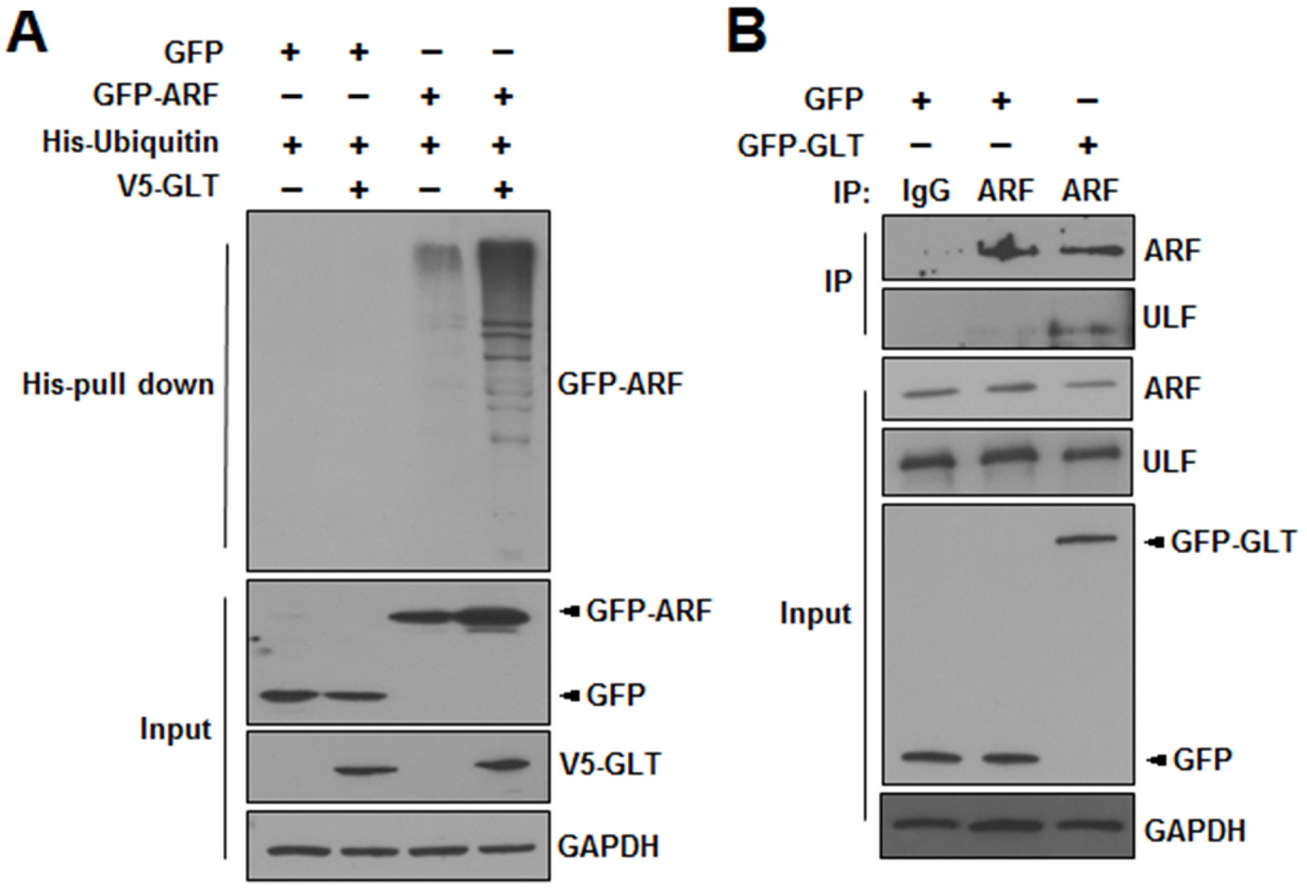
GLTSCR2 enhanced the polyubiquitination of ARF **A**. HeLa cells were transfected with the indicated plasmids for 24 h. Cells were further treated with 20 μM MG132 for 6 h, and lysates were subjected to pull-down assays using Ni^2+^-chelating sepharose. The resulting pellets were then subjected to western blotting using anti-GFP antibodies (upper panel). The expression levels of GFP or GFP-ARF, V5-GLTSCR2, and GAPDH are shown in the 3 lower panels. **B**. HeLa cells were transfected with the indicated plasmids for 24 h. Lysates were then immunoprecipitated with anti-ULF antibodies or isotype-matched control antibodies. Precipitates were subjected to immunoblotting using the indicated antibodies. The expression levels of ARF, ULF, GFP or GFP-GLT, and GAPDH are shown in the lower 4 panels.

ARF is degraded through the polyubiquitination pathway [19]. Thus, we performed ubiquitination assays to investigate whether GLTSCR2 affected ARF polyubiquitination. HeLa cells were transfected with a plasmid expressing GFP or GFP-tagged ARF (GFP-ARF) with or without plasmids expressing V5-tagged GLTSCR2 (V5-GLT) and His-ubiquitin, as indicated (Figure 6A). Interestingly, GLTSCR2 expression enhanced the polyubiquitination of ARF. ULF/TRIP12 (ULF) is a nucleoplasmic E3-ubiquitin ligase of ARF [20]; thus, the enhanced ubiquitination of ARF by GLTSCR2 could be caused by increased binding between ULF and ARF. To test this hypothesis, we performed co-immunoprecipitation and western blot analysis with HeLa cells transfected with GFP-GLT. GLTSCR2 increased the binding between ARF and ULF (Figure 6B). Thus, our results indicated that GLTSCR2 enhanced ARF degradation through the polyubiquitination pathway.

## DISCUSSION

As an oncogenic checkpoint, ARF performs several functions that are important for cell growth and death in both cancer cells and normal cells. The effects of ARF in cancer cells are largely dependent on its subcellular localization [21]. In the nucleolus, ARF assumes a stable structure by virtue of its binding with NPM and participates in ribosome biogenesis [11]. In contrast, ARF forms protein complexes in the nucleoplasm to perform various functions, such as enhancing p53 stability [22]. Nevertheless, exactly how ARF redistributes to the different locations within the cell is unclear.

Initially, we showed that GLTSCR2 bound with ARF and enhanced its release from the nucleolus. Furthermore, the binding between GLTSCR2 and ARF was essential for GLTSCR2-mediated ARF redistribution because the G3 mutant, which could not bind to ARF, failed to induce nucleoplasmic ARF redistribution. Despite these findings, how GLTSCR2 promotes nucleolar ARF release rather than sequestration, like NPM, remains unclear. Because GLTSCR2 induces the degradation of NPM, which sequesters ARF in the nucleolus, it is possible that enhanced degradation of NPM by GLTSCR2 may have the same effect [2]. However, restoring NPM expression did not significantly suppress the release of nucleolar ARF into the nucleoplasm, indicating that NPM was not involved in this process. Another model for GLTSCR2-mediated ARF release is the change in oligomerization status of ARF by binding with GLTSCR2. ARF oligomerization can influence its stability and ability to bind to HDM2 [23]. However, we observed increased ARF homodimerization when GLTSCR2 was overexpressed (data not shown). Moreover, GLTSCR2 bound to the C-terminus of ARF, while the N-terminus of ARF is essential for homodimerization [23]. Our data suggested that the redistribution of nucleolar GLTSCR2 to the nucleoplasm in a GLTSCR2-ARF complex may promote nucleoplasmic ARF translocation. This model is supported by the following observations; i) GLTSCR2-ARF binding is essential for ARF release, ii) the nucleoplasmic redistribution of GLTSCR2 in response to treatment with the JNK inhibitor SP600125 induced concomitant nucleoplasmic ARF release, and iii) the increase of nucleoplasmic GLTSCR2 by ectopic overexpression is in agreement with ARF release. Moreover, although the GLTSCR2 G2 mutant failed to cause ARF redistribution into a diffuse nucleoplasmic pattern, its expression pattern completely co-localized with that of released ARF, indicating that ARF re-localization depends on its ability to bind GLTSCR2.

In addition to nucleoplasmic redistribution, GLTSCR2 promoted ARF degradation through the polyubiquitination pathway. Nucleoplasmic ARF can be rapidly degraded through an HDM2-mediated proteasome pathway [19]. Thus, GLTSCR2 may downregulate ARF expression by enhancing nucleoplasmic translocation. The absence of a significant reduction in the half-life of ARF following G3 mutant overexpression supports the involvement of ARF translocation in the enhanced degradation process promoted by GLTSCR2. Our observations showing the increase in ULF-ARF binding supported the possibility that GLTSCR2 participated in ARF degradation through the ULF-dependent ubiquitination pathway. However, it was not clearly determined that GLTSCR2 is more actively involved in ARF degradation through the ubiquitination pathway than in inducing nucleoplasmic translocation.

In summary, GLTSCR2 was critical for promoting ARF re-localization and stability. GLTSCR2 has both oncogenic and tumor-suppressive functions, which may depend on the tissue type, expression level, subcellular distribution, and presence of binding partners [1, 2, 24]. The precise molecular mechanisms through which GLTSCR2 drives oncogenesis effects remain unclear. Thus, further studies are required to determine how the GLTSCR2-ARF axis promotes cancer.

## MATERIALS AND METHODS

### Cell culture, antibodies, and reagents

HeLa uterine cervical cancer cells and HEK-293T cells, obtained from the Korean Cell Line Bank (Seoul, Korea) and the American Type Culture Collection (Rockville, MD, USA), respectively, were cultured in Dulbecco's Modified Eagle's Medium (DMEM) with 10% fetal bovine serum and penicillin-streptomycin (Gibco, NY, USA) in a humidified incubator. HeLa cells overexpressing NPM (HeLa/NPM) were constructed by stably transfecting a plasmid encoding a V5-tagged variant of the NPM protein (pcDNA-NPM/V5) into HeLa cells, followed by G418 selection for 4 wks. Rabbit polyclonal antibodies against GLTSCR2 were prepared as described previously [25]. Anti-ARF, anti-GFP, anti-GAPDH, and anti-GST antibodies were purchased commercially from Cell Signaling Technology, Inc. (Danvers, MA, USA). Unless otherwise specified, all other reagents were obtained from Sigma-Aldrich, Inc. (St. Louis, MO, USA).

### Plasmid, transfection, and GLTSCR2 knockdown

Plasmids for wild-type or mutant forms of GLTSCR2 and ARF were generated by the polymerase chain reaction (PCR) and standard cloning techniques, as previously described [25]. Construction of a doxycycline-inducible (Tet/Off system) adenovirus expressing GFP-tagged GLTSCR2 was described previously [15]. Cells were transfected with plasmids using Lipofectamine 2000 (Invitrogen, Carlsbad, CA, USA) according to the manufacturer's recommendations. The expression of GLTSCR2 was blocked by transfection with siRNA targeting GLTSCR2 (Qiagen Inc., CA, USA) using Oligofectamine (Invitrogen).

### Adenoviral transduction

Adenoviral transduction of HeLa cells was performed according to the manufacturer's recommended protocol (Adeno-X Tet-Off Expression System 1, Clontech). Briefly, cells were seeded at a density of 3 × 10^5^ cells/6-cm dish in Opti-MEM (GibcoBRL) and exposed to adenovirus expressing GLTSCR2 and a regulatory adenovirus at a multiplicity of infection (MOI) of 250 for 4 h. Then, freshly prepared DMEM containing 20% FBS was added. The transduction efficiency was determined by fluorescence microscopy.

### Recombinant proteins and pull-down assays

One microgram of recombinant GST fusion protein was immobilized on glutathione-sepharose beads in phosphate-buffered saline (PBS) for 30 min at 4°C and then mixed with lysates from HEK-293T cells after transfection with the indicated plasmids. The samples were then centrifuged and washed with cold PBS 4 times. The bound proteins were eluted by boiling and then detected by western blotting. Recombinant ARF-GST fusion protein was produced by transforming *E. coli* with the pGEX2TK-ARF vector, induction with IPTG, and purification using a column packed with glutathione resin (Thermo Fisher Scientific Inc., NY, USA). Recombinant GLTSCR2-GST protein was obtained from Abnova Corporation (Taipei, Taiwan).

### Immunoblotting, immunocytochemistry, and immunoprecipitation

Immunoblotting was performed as described previously [1]. For immunostaining, pretreated cells were fixed with 4% paraformaldehyde and incubated sequentially with primary antibodies overnight at 4°C and secondary antibodies for 2 h at 4°C. Cells were viewed under an inverted confocal microscope (META 510; Zeiss, Germany) after nuclear staining with 4′,6-diamidino-2-phenylindole (DAPI). For immunoprecipitation, cells were lysed in lysis buffer (500 mM NaCl, 50 mM Tris-HCl pH 7.5, 0.5% Triton X-100, 1 mM EDTA, and 1 μM DTT), clarified by centrifugation, incubated with the indicated antibodies, and immunoprecipitated with protein A (GE Healthcare). The precipitates were washed 4 times, subjected to sodium dodecyl sulfate polyacrylamide gel electrophoresis, and analyzed by western blotting.

### PLA experiments

PLAs were performed using a Duolink II Detection Kit (Olink Bioscience, Uppsala, Sweden) according to the manufacturer's protocols. Briefly, cells were incubated with rabbit anti-GLTSCR2 and mouse anti-ARF antibodies, after which secondary antibodies conjugated with oligonucleotides (PLA probe anti-mouse MINUS and PLA probe anti-rabbit PLUS) were added. Ligation and amplification were performed using a rolling circle amplification step. The signal was visualized as an individual fluorescent spot. The spots were counted from at least 100 cells.

### Ubiquitination assay

His-tagged ubiquitin and plasmids expressing either V5-GLTSCR2 or GFP-ARF were transfected into HeLa cells for 24 h. The cells were then treated with MG132 (20 μM) for 6 h to inhibit proteasomal degradation and then harvested in lysis buffer (6 M guanidine-HCl, 0.1 M NaPi, 10 mM Tris-HCl [pH 8.0], 5 mM imidazole, 10 mM β-mercaptoethanol). The lysates were then incubated with Ni^2+^-chelating sepharose for 3 h at room temperature and washed 5 times with a buffer composed of 8 M urea, 0.1 M NaPi, 10 mM Tris-HCl (pH 6.3), and 10 mM β-mercaptoethanol.

### Statistical analysis

Statistical analysis was performed using SPSS software, version 13.0 (SPSS, Chicago, IL, USA). Data were analyzed with Student's t test. Differences with *p* values of less than 0.05 were considered statistically significant.

## SUPPLEMENTARY MATERIALS FIGURES AND TABLES



## Abbreviations

ARF: alternative reading frame
GLTSCR2: glioma tumor-suppressor candidate region gene 2 protein
NPM: nucleophosmin

## Abbreviations

ARF: alternative reading frame
GLTSCR2: glioma tumor-suppressor candidate region gene 2 protein
NPM: nucleophosmin
