# Supplementary material for: GLTSCR2 promotes the nucleoplasmic translocation and subsequent degradation of nucleolar ARF
Source: Oncotarget. 2016 Apr 28;8(10):16293–302. doi: 10.18632/oncotarget.9957 (PMC5369963; doi:10.18632/oncotarget.9957)
Supplement: Supplementary file 1 [file oncotarget-08-16293-s001.pdf]

GLTSCR2 promotes the nucleoplasmic translocation and subsequent degradation of nucleolar ARF

SUPPLEMENTARY FIGURES

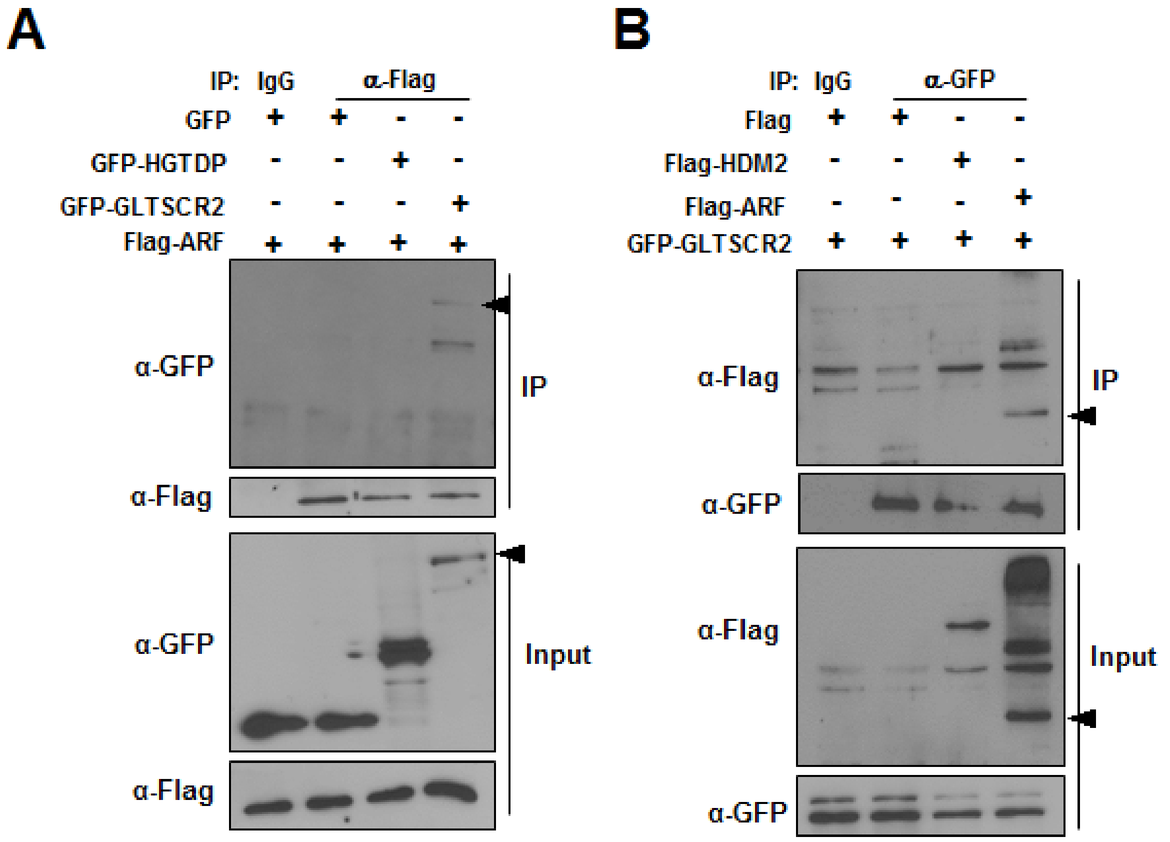

Supplementary Figure S1: A. HEK-293T cells were transfected with the indicated plasmids for 24 h, and cell lysates were immunoprecipitated using a control IgG or an anti-Flag antibody. Precipitates were subjected to western blot analysis. **B.** Cells were transfected with the indicated plasmids for 24 h, and cell lysates were immunoprecipitated using a control IgG or an anti-GFP antibody. Precipitates were subjected to western blot analysis.

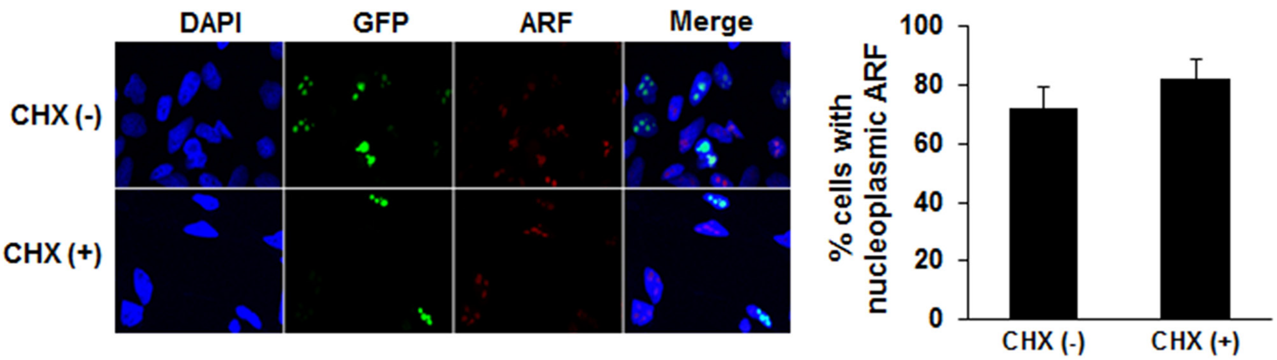

Supplementary Figure S2: Cells were transduced with Ad-GLT for 12 h, after which they were left untreated or were treated with 100 µg/ml cycloheximide for an additional 4 h, followed by immunocytochemical staining for ARF. Representative images are shown in the left panel. Cells with nucleoplasmic ARF were counted among at least 200 cells following Ad-GLT transduction under a fluorescence microscope (right panel). No statistically significant difference was observed between the 2 groups.

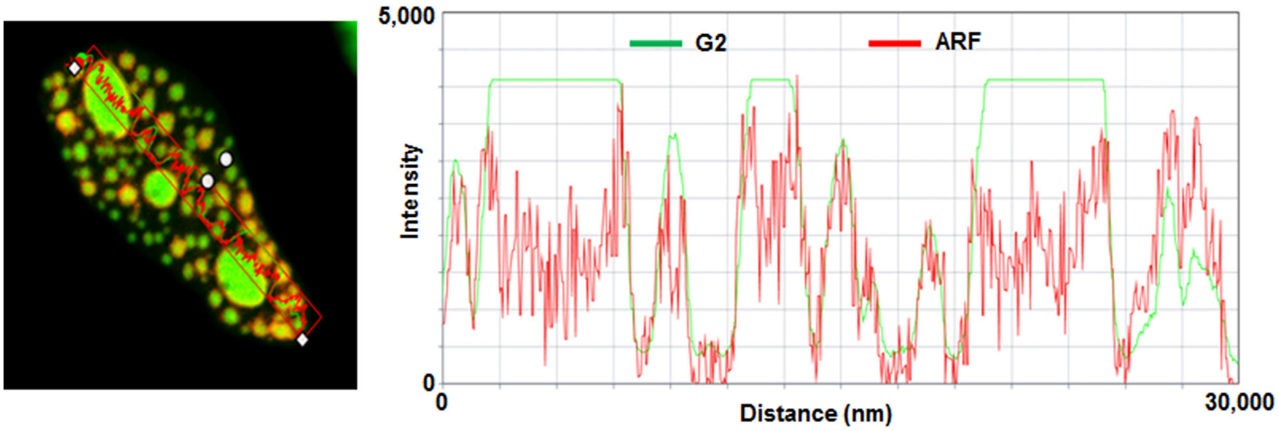

Supplementary Figure S3: A plasmid encoding theG2 mutant of GLTSCR2 was transfected into HeLa cells. After 24 h, immunostaining was performed using an anti-ARF antibody, and the fluorescence-intensity profile was assessed using ZEN software (Carl Zeiss Microscopy GmBH, Germany).

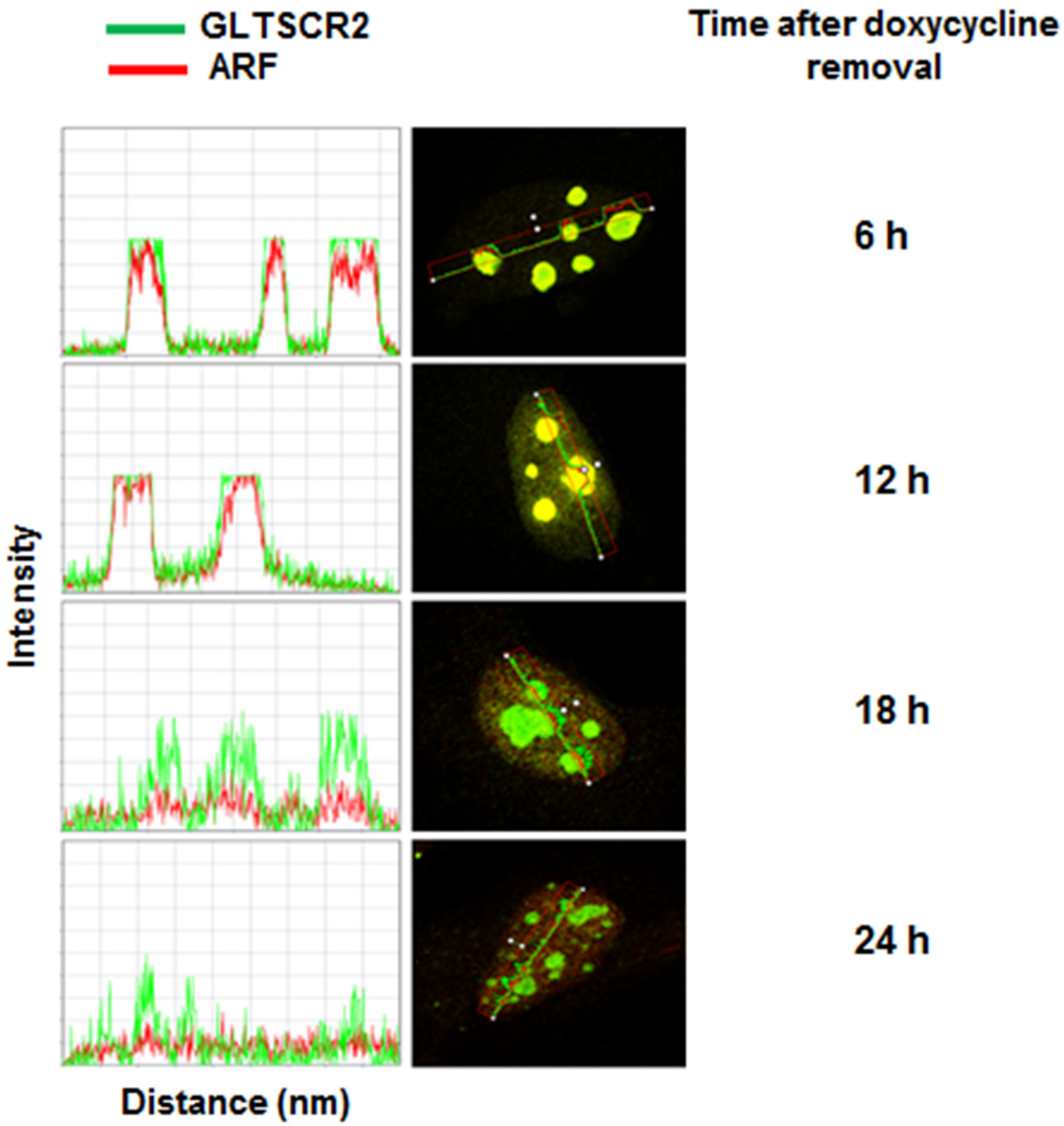

Supplementary Figure S4: Assessment of the fluorescence-intensity profile in cells shown in Figure 4C, using ZEN software.

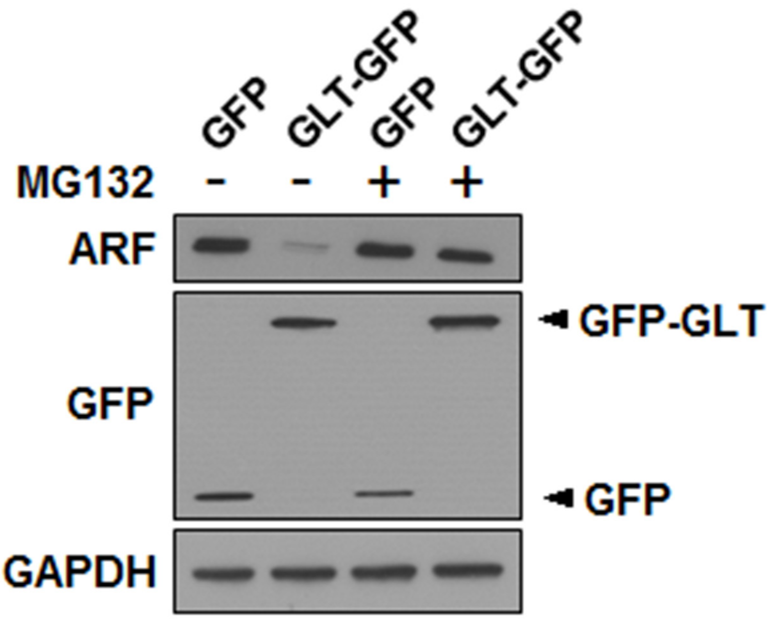

Supplementary Figure S5: Cells were transduced with Ad-GLT or Ad-GFP for 24 h, after which they were left untreated or were treated with 20 μM MG132 for additional 6 h, as indicated. Lysates were subjected to western blot analysis using anti-ARF and anti-GFP antibodies, and relative expression was determined after normalization to GAPDH expression.

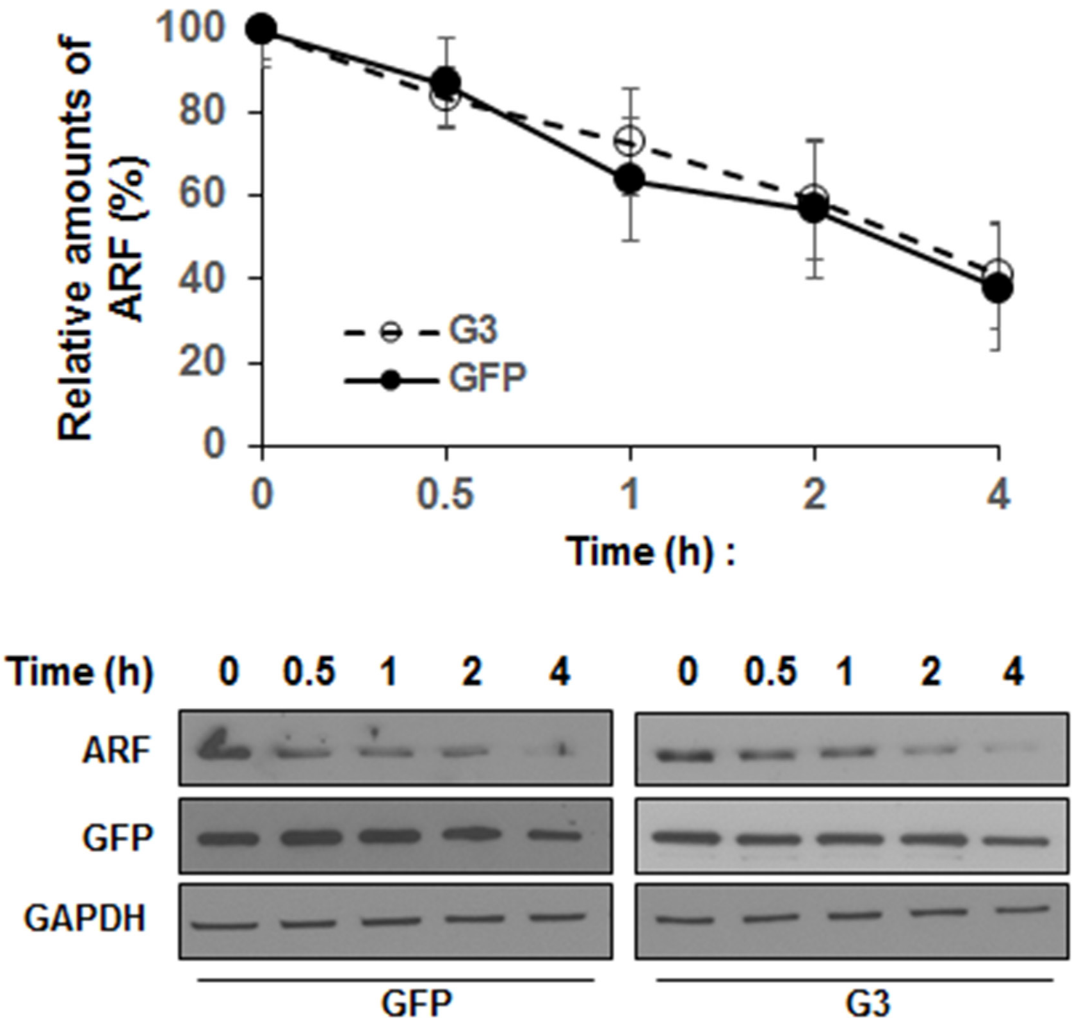

Supplementary Figure S6: HeLa cells were transfected with a plasmid expressing the G3 mutant (G3) or an empty control plasmid (GFP) for 24 h. After treatment with 100 µg/mL cycloheximide, cell lysates were prepared after 0–4 h. ARF expression was determined by immunoblotting and normalization to GAPDH expression. The plot shows the densitometric quantification of the results from cycloheximide chase assays (upper panel). The data shown represent the percentages of ARF intensity from 3 independent experiments, compared with that at the 0-h time point. Representative immunoblot images of cycloheximide chase assays are shown in the lower panel.
